# Comparison of the impact of longitudinal body mass index changes on cardiac arrest risk between normal and overweight populations

**DOI:** 10.1002/jcsm.13504

**Published:** 2024-06-18

**Authors:** Youn‐Jung Kim, Min‐Ju Kim, Ye‐Jee Kim, Won Young Kim

**Affiliations:** ^1^ Department of Emergency Medicine Asan Medical Center, University of Ulsan College of Medicine Seoul South Korea; ^2^ Department of Clinical Epidemiology and Biostatistics Asan Medical Center Seoul South Korea

**Keywords:** body mass index, body weight, obesity, out‐of‐hospital cardiac arrest, overweight

## Abstract

**Background:**

Being overweight is a key modifiable risk factor for cardiovascular disease. However, the impact of longitudinal changes in body mass index (BMI) on the risk of out‐of‐hospital cardiac arrests (OHCA) remains unclear, especially among overweight populations.

**Methods:**

This nested case–control study utilized data from the Korean National Health Information Database between 2009 and 2018. A total of 23 453 OHCA patients, who underwent national health check‐ups within 1 and 2–4 years before OHCA occurrence, and 31 686 controls, who underwent similar national health check‐ups, were included. The study population was matched for sex, age and survival status. Conditional logistic regression was employed to analyse the odds ratios (ORs) and 95% confidence intervals (CIs) of each BMI per cent change in assessing the risk of OHCA occurrence within 1 year.

**Results:**

A reverse J‐shaped association between BMI per cent change and OHCA risk was observed, even among overweight populations. Among the overweight populations, weight loss significantly increased OHCA risk, with ORs (95% CI) of 4.10 (3.23–5.20) for severe weight loss (BMI decrease > 15%), 2.72 (2.33–3.17) for moderate weight loss (BMI decrease 10–15%) and 1.46 (1.35–1.59) for mild weight loss (BMI decrease 5–10%). Conversely, mild weight gain (BMI increase 5–10%) did not significantly increase OHCA risk. The impact of weight changes on the occurrence of OHCA differed by sex, being more prominent in males.

**Conclusions:**

Significant weight changes within a 4‐year period increase the risk of OHCA with a reverse J‐shaped association, even among overweight and obese individuals. Maintaining a stable weight could be a reliable public health strategy irrespective of the weight status, particularly for males.

## Introduction

Considering the dismal outcomes of out‐of‐hospital cardiac arrests (OHCA), identifying individuals at high risk and implementing prevention strategies could contribute to a lower incidence of cardiac arrests and ultimately enhance public health. Being overweight is the second‐leading modifiable risk factor for cardiovascular disease,^1^ significantly contributing to cardiovascular risk factors such as hypertension, diabetes and dyslipidaemia, which are associated with an elevated risk of cardiac arrest.^2^

Previously, we found that significant weight changes increase the risk of OHCA within a year, highlighting the importance of maintaining a stable weight to reduce the risk of cardiac arrest.^3^ However, the dynamic changes in body mass index (BMI) in the overweight population and their associated risk of cardiac arrest remain unknown. This population‐based nested case–control study aimed to compare the impact of longitudinal BMI changes on cardiac arrest occurrence between normal and overweight populations, utilizing data from national healthcare insurance and health screening examinations.

## Methods

This population‐based nested case–control study utilized claims data from the Korean National Health Information Database, collected between 2009 and 2018. Cases were adults who received cardiopulmonary resuscitation at an emergency department due to non‐traumatic OHCA between 2010 and 2018 and who underwent national health check‐ups twice: once within a year and another within 2–4 years before OHCA. Controls were adults without a history of OHCA who underwent similar health check‐ups. Cases and controls were matched at a 1:2 ratio based on sex, age and alive status at the time of cardiac arrest.

Body weight and height data were extracted from the national health check‐up results conducted within 1 and 2–4 years before OHCA, respectively. The BMI was calculated as weight in kilograms divided by the square of height in metres (kg/m^2^). After excluding participants with underweight status, individuals were categorized into four groups based on their BMI according to the World Health Organization criteria for Asian populations: normal weight (18.5–22.9 kg/m^2^); overweight (23.0–24.9 kg/m^2^); class I obesity (25.0–29.9 kg/m^2^); and class II obesity (≥30.0 kg/m^2^).^3^ Conversely, for Western countries, a BMI range of 18.5–24.9 kg/m^2^ is considered normal weight; 25.0–29.9 kg/m^2^ is considered overweight; and ≥30.0 kg/m^2^ is considered obese. Excluding the group with a small number of patients in the class II obesity category, we compared the impact of longitudinal BMI changes on cardiac arrest risk between the normal‐weight group and the overweight group, defined as including both the overweight and class I obesity categories.

For assessing longitudinal BMI changes, the per cent change in BMI was calculated using the formula: (BMI_within a year_ − BMI_previous 4 years_)/BMI_previous 4 years_ × 100. Stable weight change was defined as the absolute value of BMI per cent change being <5%, and a BMI per cent change exceeding 5% was considered a significant change. Seven groups were defined based on per cent change around these thresholds: (1) severe weight loss (BMI decrease over 15%), (2) moderate weight loss (10–15% BMI decrease), (3) mild weight loss (5–10% decrease), (4) stable weight (absolute value of BMI per cent change < 5%), (5) mild weight gain (5–10% increase), (6) moderate weight gain (10–15% increase) and (7) severe weight gain (BMI increase over 15%).^3^ We utilized conditional logistic regression with Enterprise Guide (Version 7.1; SAS Institute Inc., Cary, NC, USA) to estimate the odds ratios (ORs) and 95% confidence intervals (CIs) of each variable in assessing the risk of OHCA occurrence within a year.

## Results

Among the 23 453 non‐traumatic adult OHCA cases and 31 686 control participants, the mean age was 65 years, and 71.9% were male. The participants were categorized into three groups based on their previous weight status: the previous normal‐weight group (35.2%), the overweight group (61.2%) and the class II obesity group (3.5%). *Table* *1* summarizes the baseline characteristics between OHCA cases and controls. Over the study period, a BMI per cent change of more than 5% decrease was observed more frequently in OHCA cases than in the control group. Individuals previously categorized as normal weight exhibited a higher frequency of significant BMI decreases in OHCA cases, while stable weight was more prevalent among the controls. Similar trends were observed in patients previously classified as overweight.

**Table 1:** Baseline characteristics and body mass index per cent changes according to the previous weight status between out‐of‐hospital cardiac arrest and control cases

Characteristics	Total (*N* = 55 139)	OHCA case (*N* = 23 453)	Controls (*N* = 31 686)	*P*‐value
Age, years	65.0 ± 11.2	65.0 ± 11.6	65.0 ± 11.2	
Male	39 624 (71.9%)	16 900 (72.1%)	22 724 (71.7%)	
BMI status				<0.001
Previous normal‐weight group	19 411 (35.2%)	8653 (36.9%)	10 758 (34.0%)	
Previous overweight group	33 770 (61.2%)	13 786 (58.8%)	19 984 (63.1%)	
Previous class II obesity group	1953 (3.5%)	1009 (4.3%)	944 (3.0%)	
BMI per cent changes according to the weight status				
Previous normal‐weight group	*N* = 19 411	*N* = 8653	*N* = 10 758	<0.001
Stable	11 825 (60.9%)	4855 (56.1%)	6970 (64.8%)	
Significant BMI decrease	3092 (15.9%)	1775 (20.5%)	1317 (12.2%)	
Significant BMI increase	4499 (23.2%)	2028 (23.4%)	2471 (23.0%)	
Previous overweight group	*N* = 33 770	*N* = 13 786	*N* = 19 984	<0.001
Stable	23 004 (68.1%)	8594 (62.3%)	14 410 (72.1%)	
Significant BMI decrease	6410 (19.0%)	3353 (24.3%)	3057 (15.3%)	
Significant BMI increase	4356 (12.9%)	1839 (13.3%)	2517 (12.6%)	
Previous class II obesity group	*N* = 1953	*N* = 1009	*N* = 944	<0.001
Stable	1169 (59.9%)	569 (56.4%)	600 (63.6%)	
Significant BMI decrease	577 (29.5%)	344 (34.1%)	233 (24.7%)	
Significant BMI increase	207 (10.6%)	96 (9.5%)	111 (11.8%)	

*Note*: The participants were categorized into three groups based on their BMI: normal weight (18.5–22.9 kg/m^2^); overweight, defined as including both the overweight (23.0–24.9 kg/m^2^) and class I obesity (25.0–29.9 kg/m^2^); and class II obesity (≥30.0 kg/m^2^) groups according to the World Health Organization criteria for Asian populations. Abbreviations: BMI, body mass index; OHCA, out‐of‐hospital cardiac arrest.

**Figure 1:**
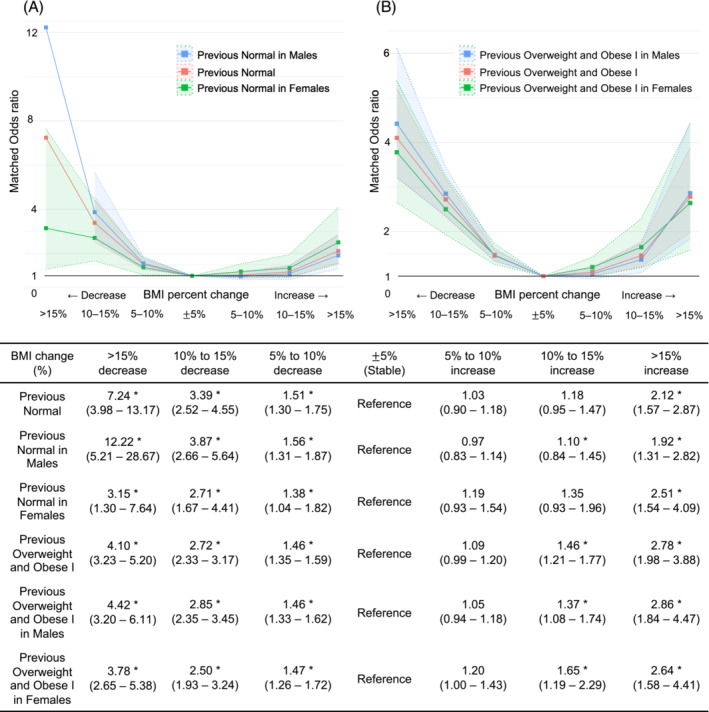
Association between per cent changes in body mass index (BMI) and out‐of‐hospital cardiac arrest: (A) normal‐weight population and (B) overweight population. *Statistically significant at *p* ⟨〈 0.05.

The association between OHCA occurrence and BMI per cent change varied by previous weight status and sex (*Figure* *1*). The overall pattern of association was reverse J‐shaped, showing that even mild weight loss (a BMI decrease of 5% or more) significantly increased OHCA risk, unlike weight gain. The effect of weight changes on OHCA risk varied based on previous weight status. In normal‐weight individuals, weight loss significantly increased OHCA risk, with ORs (95% CI) of 7.24 (3.98–13.17) for severe weight loss (BMI decrease > 15%), 3.39 (2.52–4.55) for moderate weight loss (BMI decrease 10–15%) and 1.51 (1.30–1.75) for mild weight loss (BMI decrease 5–10%) (*Figure* *1* *A*). Conversely, only severe weight gain (BMI increase > 15%) showed a significant increase in OR (95% CI) of 2.12 (1.57–2.87). The effect of weight loss on increasing OHCA risk remained prominent in participants with previous overweight and class I obesity status (*Figure* *1* *B*). Mild weight loss (BMI decrease 5–10%) increased the OHCA risk, reporting an overall OR of 1.46, comparable to the impact of moderate weight gain (BMI increase 10–15%) with an overall OR of 1.46. Additionally, the association between weight changes and OHCA risk differed by sex, being less pronounced in females than in males.

## Discussion

The findings of our study showed no significant difference in the impact of longitudinal BMI changes on cardiac arrest occurrence between overweight and normal‐weight populations. Maintaining weight stability (BMI per cent change < 5%) or experiencing mild weight gain (BMI per cent change of 5–10%) appears to be the most favourable strategy, associated with no significant increased risk of OHCA, regardless of weight status. The impact of significant body weight changes on OHCA risk is more pronounced in males, suggesting that they are more vulnerable to weight changes, especially weight loss, than females.

We observed a reversed J‐shaped relationship between weight loss and weight gain, associated with an increased OHCA risk, particularly among overweight and obese individuals. These findings indicate that weight loss may be a more unfavourable change than weight gain, even in obese individuals, contrary to common belief. A previous study reported a stronger association between weight loss and increased mortality risk than weight gain in patients undergoing cardiac catheterization.^4^ The association may initially seem to be due to unintentional weight loss related to their comorbid diseases. However, weight changes still demonstrated a reverse J‐shaped curve for all‐cause mortality, independent of BMI status, in other population studies, even after adjusting for confounding factors related to weight changes.^5^ Moreover, another epidemiologic study reported increased non‐cancer mortality rates in overweight or obese individuals after weight loss.^6^ Different changes in body composition could be alternative explanations.^4^, ^7^ Metabolic benefits from weight loss mainly originate from the reduction of visceral adipose tissue, and modest weight loss shows the most significant preference for losing visceral adipose tissue compared with subcutaneous abdominal fat.^8^ Overt weight loss may contribute more to muscle mass decline associated with frailty than a reduction in fat mass.^4^, ^7^ Additionally, weight gain is a natural change over the life span, which may explain why people showed weight tolerance to weight gain compared with weight loss.^9^

The sex dimorphism in the association between weight loss and increased OHCA risk is also a notable finding of our study. Our findings suggest that males are more sensitive to the effects of weight loss compared with females. While this association was more pronounced in individuals with normal weight status, it was consistently observed in those with overweight and class I obesity. A recent large randomized controlled dietary intervention study highlighted distinct sexual dimorphism in overweight or obese individuals.^10^ Males exhibited more significant weight loss and improvements in cardiometabolic risk parameters, such as plasma lipids and insulin resistance, following an 8‐week low‐calorie diet; however, they also experienced a greater weight regain and a rebound in these profiles during a 6‐month maintenance period.^10^ In addition to differences in body fat distribution and metabolic status, higher body weight fluctuations in males could contribute to their elevated ORs for OHCA linked to weight changes.^10^

This study has some limitations. Firstly, despite conducting a stratified analysis by weight status and sex, it was not possible to adjust for various potential confounding factors, such as comorbid diseases, diet and exercise. Secondly, the specific causes of OHCA remained unknown. Thirdly, this study only included Koreans, and the association may vary across different ethnicities. Lastly, the underlying reasons for weight changes in individuals remained unknown.

In conclusion, our findings highlight a reverse J‐shaped association between significant weight changes within a 4‐year period and the occurrence of OHCA, even among overweight and obese individuals. This association was particularly pronounced in males. The study emphasizes the importance of maintaining a stable weight as a reliable public health strategy to prevent OHCA occurrence, irrespective of one's weight status.

## Conflict of interest statement

The authors declare that they have no competing interests.

## Funding

This research was supported by a research grant from Medical AI Co., Ltd.
